# Leave of Absence

**Published:** 1873-07

**Authors:** 


### LEAVE OF ABSENCE.

WE fail to see the use of seeking it across seas, oceans and continents—shattered on the rail, bumped on the billows, sleeping on shelves, eating you know not what, prepared in smoked and cobwebbed kitchens, with hands of a semicircle of black or red finger ends; the tough meat, the soggy bread, the sour cream and the watery potatoes; to get into bed at midnight, to be waked up before day; no broad hair mattress to sleep upon; no high, and airy, and capacious chamber; no beloved Croton, hot and cold; no porter house steaks, no broiled chicken, no delicious shad, no ice cream, no home made bread, no snow-white table-cloth, no clean napkin, "no nothin'." What's the use of all this? To exchange the quiet of one's own home, in the broad and beautiful street, away from the rumble of the omnibus and the screech of the locomotive, for the noise, and hubbub, and clatter of hotels! Only think of it: to be cooped up in a room day and night, week in and week out, with scarcely space enough for an extra chair, a damp towel, and a quart of water for washing; mosquitoes by night and flies by day, and night and day the fumes of kitchen cookery filling alike the chamber and the parlor. If you want to take a walk there is dampness in the grass, and there is dust in the road, and fierce sunshine everywhere, making your room like an oven. Instead of having the range of the whole house, to go where and when you please, and when tired of one apartment go to another—when weary of up stairs you can go down, and feel at home everywhere—you have but one single apartment, two yards long and no yards broad, and, "like as not," at the top of the house, immediately under a scorching roof. This is called spending the summer in the country, with its delightful visions of pure milk, fresh laid eggs and home made bread, topped off with spring chickens and strawberries and cream.

In view of all this we have concluded, against our wish—for we desire no better place to live in than New York during our mundane state, and never expect to find one as good—to cross the great sea again, and with wife and three daughters to see the sights of the Old World. First and foremost, with the "Satchel Guide to Europe" in hand, we'll make acquaintance with "Paddy from Cork, with a pocket behind," to take us to pay our respects to the Blarney Stone and the Lakes of Killarney—on to famous Dublin, and Belfast, and the Giant's Causeway; thence to Glasgow, "Edinboro' town" and Abbotsford, with all its memories; not forgetting, at Glasgow, to give a day to the "Banks and Braes o' Bonnie Doon." But time and space would fail to speak of London, and Paris, and Heidelberg, and the Rhine, and Berlin, and Vienna, and Switzerland, and Florence, and ever so many other places. Maybe we shall tell our readers of some of them in the course of the season. Still, if we had our way, we would stay in New York all summer, and quietly read for ourselves and write for others. What's the use of the "country?" It was made for farm people to raise potatoes, and grow cabbages, and "all them things." It does well enough, now and then, to take a ride out there, provided you can get back home by sundown, and get rid of the barking of dogs and the cackling of geese. There are more flies in the country than anything else; they won't let you sleep in the morning, they won't let you doze in the day, they drop in your coffee at breakfast, they float on your soup at dinner, they crawl in your gravy, they wriggle in your preserves and mix in your pies. Bah! The fact is, we will sell out all our stock in trade of all the "country" between this and the North Pole for two cents. One leisure stroll down Broadway is worth all the "roses and posies" in the land. There is a great deal of talk about the "breezes and the treeses"—the plenty of the one and cooling shades of the other; but the breezes blow gnats, and under every shade there are bushels of bugs and creeping things, whose very crawl makes your flesh creep to think of. Let the reader just think of one single fact in connection with country life and "country seats"—you can buy a million of them any day, but it will take you five years to sell one off your hands. This single, simple, indisputable fact tells the whole story of the delights of a country life.

